# Mechanical Testing and Modeling of the Time–Temperature Superposition Response in Hybrid Fiber Reinforced Composites

**DOI:** 10.3390/polym13071178

**Published:** 2021-04-06

**Authors:** Aggelos Koutsomichalis, Thomas Kalampoukas, Dionysios E. Mouzakis

**Affiliations:** 1 Mechanics of Materials and Engineering Lab, Hellenic Airforce Academy, Tatoion Airforce Base, GR13672 Acharnes, Greece; a.koutsomichalis@gmail.com (A.K.); tomikala@hotmail.com (T.K.); 2 Mechanics of Materials Laboratory, Sector of Mathematics and Engineering Applications, Hellenic Army Academy, GR16672 Vari, Attika, Greece

**Keywords:** composites, time–temperature superposition analysis, hybrid fabrics, viscoelastic response, creep, flexural failure, dynamic mechanical analysis

## Abstract

The purpose of this study was to manufacture hybrid composites from fabrics with superior ballistic performance, and to analyze their viscoelastic and mechanical response. Therefore, composites in hybrid lay-up modes were manufactured from Vectran, Kevlar and aluminum fiber-woven fabrics through a vacuum assisted resin transfer molding. The specimens were consequently analyzed using static three-point bending, as well as by dynamic mechanical analysis (DMA). Apart from DMA, time–temperature superposition (TTS) analysis was performed by all available models. It was possible to study the intrinsic viscoelastic behavior of hybrid ballistic laminates, with TTS analysis gained from creep testing. A polynomic mathematical function was proposed to provide a high accuracy for TTS curves, when shifting out of the linearity regimes is required. The usual Williams–Landel–Ferry and Arrhenius models proved not useful in order to describe and model the shift factors of the acquired curves. In terms of static results, the highly nonlinear stress–strain curve of both composites was obvious, whereas the differential mechanism of failure in relation to stress absorption, at each stage of deformation, was studied. SEM fractography revealed that hybrid specimens with Kevlar plies are prone to tensile side failure, whereas the hybrid specimens with Vectran plies exhibited high performance on the tensile side of the specimens in three-point bending, leading to compressive failure owing to the high stress retained at higher strains after the maximum bending strength was reached.

## 1. Introduction

Composite materials are very attractive for the aerospace, automotive, defense and wind power industries due to their lower density, higher stiffness, higher strength, and better fatigue resistance compared to metals [1]. The use of hybrid composite laminates can lead to interesting properties owing to the combination of reinforcement materials [2]. It was found that by increasing the hybrid ratio of laminates reinforced with high modulus polyimide and carbon fibers, in an epoxy resin matrix, that a higher tensile modulus can be achieved, whereas the tensile failure strength is decreased [3]. Furthermore, by studying the glass-carbon fiber hybrid composites pipes, it was found that the same properties of the single carbon fiber composite could be obtained by using the hybrid composites, thus resulting in a cost reduction [4]. For example, a study of eco-friendly hybrid composites with banana and sisal reinforcements concluded, that a significant loss of mechanical strength occurred when compared to the composites with single type of reinforcement [5]. In contrast, Kevlar/epoxy resin composite reinforced with nano-silica particles possessed superior mechanical properties compared with Kevlar composites [6]. Hybrid Kevlar composites with basalt natural fibers as a second reinforcement was also studied [7]. As the effect of glass fiber reinforcement to Kevlar/epoxy resin composites was studied before [8,9], the authors were not able to locate any other study of Vectran-glass hybrid composite, or a direct comparison of thermomechanical response in the two hybrid laminates.

One of the tests used to assess damage initiation and propagation in fiber composites is the three-point beam test. The use of the three-point flexural test has been used either for estimating the effect of various parameters such as fiber orientation, laminate stacking, surface waviness and molding temperature to the composite flexural behavior [10,11], or for analyzing the failure mode [12,13]. Three-point beam impact tests on fiber composites are often performed, and their results show that the consistent values are elastic deflection to failure, and hence peak impact force. Once the peak force has been reached, failure normally initiates through fiber fracture at the opposite of the force face [14].

In order to determine the mechanical properties of laminated composite materials, various specimen configurations with different laminate arrangements can be used and subjected to static loading. In the case of unidirectional laminated composites, the mechanical properties can be divided into two main categories: normal and shear. Concerning in-plane or intralaminar shear properties, there exists a variety of different shear test methods depending on the specimen’s geometry, the type of applied load and the laminate configuration [15,16,17,18,19,20].

In this study, three-point bending of the laminates’ composite structure was investigated. To characterize the bending loads in composite laminate structures, they were subjected to three-point bend testing. The load-displacement curves were obtained to characterize the failure mechanisms in the hybrid composite laminates, and scanning electron microscope photos were obtained to characterize the failure mechanisms in the composite laminate, by considering the effects of stacking sequences used. The thermomechanical response of these materials was studied by means of dynamic mechanical analysis. Time–temperature superposition (TTS) analysis was performed on creep curves, and a polynomic function was used to simulate the shift-factors beyond the applicability regime of the usual viscoelastic theoretical models.

## 2. Materials and Experimental Procedures

### 2.1. Composite Materials

Fiberglass aluminum fabric along with aramid ballistic fabric and Vectran fabric (a high performance thermoplastic multifilament yarn spun from Vectran^®^ (Kuraray, Tokyo, Japan) liquid crystal polymer) were used to manufacture composite laminated plates (Table 1 lists their properties). A high performance epoxy laminating system with slow hardener was used for the preparation of the hybrid composite.

**Figure 1:**
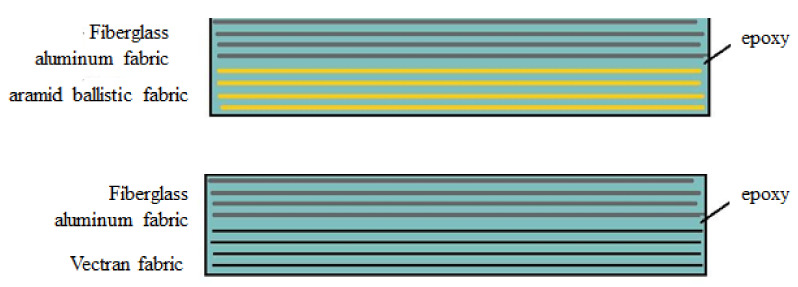
Lay-up of the hybrid composite specimens.

Each plate had dimensions of 40 × 40 cm and consisted of four layers of fiberglass aluminum fabric along with aramid ballistic fabric and Vectran fabric, respectively, as shown in Figure 1.

The resin type chosen as a matrix, was the high-performance epoxy system comprising Epoxy resin R2930 and the slow hardener H3033, both obtained from Fibermax Ltd (London, UK), with typical properties up to 85 MPa tensile strength, 3.35 GPa tensile modulus, fracture strain of 7.5% and a glass transition temperature of 100 °C.

### 2.2. Hybrid Laminates Fabrication

Laminate fabrication, which consisted of two different stacking sequences of four layers each, was prepared by hand lay-up, and vacuum molding process. The pressure was 0.1 MPa, curing took place at room temperature for 24h, and post-curing was conducted at 80 °C for 8 h.

### 2.3. Three-Point Bending Test

**Figure 2:**
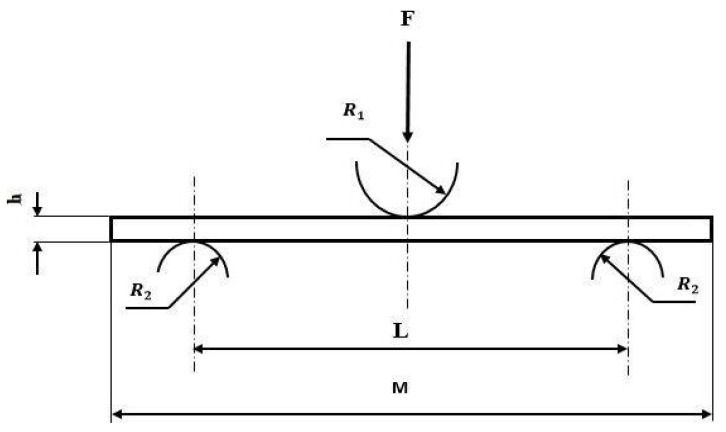
Load configuration for a beam in three-point bending test.

The three-point bending tests were conducted with an Instron 5940 universal testing machine, per EN-ISO 178:1996. The applied velocity of the bending load was 2 mm/min. Figure 2 shows the loading configuration for a beam in a three-point bending test, where F is the applied force, R1 is the indenter radius, R2 is the fixed support radius, h is the specimen thickness, L is the support span and M is the specimen length. Load-displacement plots were obtained for each test specimen. Specimen dimensions were approximately 50 × 15 × 2.5 mm^3^.

The flexural stress is given by the following equation

*σ_f_* = (3*FL*/*2bh*^2^) (1)

where *σ_f_*, is the flexural stress (MPa), F is the applied load (N), *L* is the span (mm), *h* is the thickness of the specimen (mm) and *b* is the width of the specimen (mm).

### 2.4. Thermomechanical Testing and Modeling

All thermomechanical testing (dynamic mechanical analysis (DMA) and creep testing) was performed on a TA Instruments, DMA Q800 DMA Instrument (Delaware, DE, USA). The dynamic thermomechanical testing was performed on composite specimens in three-point bending mode which had dimensions of approximately 60 × 12 × 3.5 mm^3^. The experimental conditions involved a thermal stepping of 3 °C, isothermal conditioning for 3 min, which was followed by a frequency sweep of 1, 2, 5, 10, 20, 50, and 100 Hz at every thermal step. The procedure was performed for temperatures of 25 to 250 °C. Maximum oscillation amplitude was set at 1N, and static force was kept constant at 0.0010 N, respectively.

Time–temperature superposition (TTS) analysis was performed on specimens under the same loading type and dimensions as the ones in thermomechanical testing. Creep testing TTS protocol was applied: temperature was ramped from 25 to 250 °C at a step of 5 °C. A 3 min isothermal pause was then applied at each step. Creep stress was set at 1 MPa for 10 min, before temperature was stepped again. In this way, the main creep-bending TTS data were collected.

Analysis of the experimental data was performed with software suites provided by TA Instruments namely: “Universal Analysis” and “Data Analysis” respectively.

### 2.5. Scanning Electron Microscopy

Scanning electron microscopy (SEM) analysis of the specimens, tested under three-point bending, was performed on a JSM-7610F SEM from JEOL (Tokyo, Japan). JSM-7610F, an ultra-high-resolution Schottky Field Emission Scanning Electron Microscope, which has semi-in-lens objective lens. High power optics can provide high throughput and high-performance analysis. Acceleration Voltage was set at 1 kV, and specimens were at their natural state without any sputtering.

## 3. Results & Discussion

### 3.1. Three-Point Bending Test

**Figure 3:**
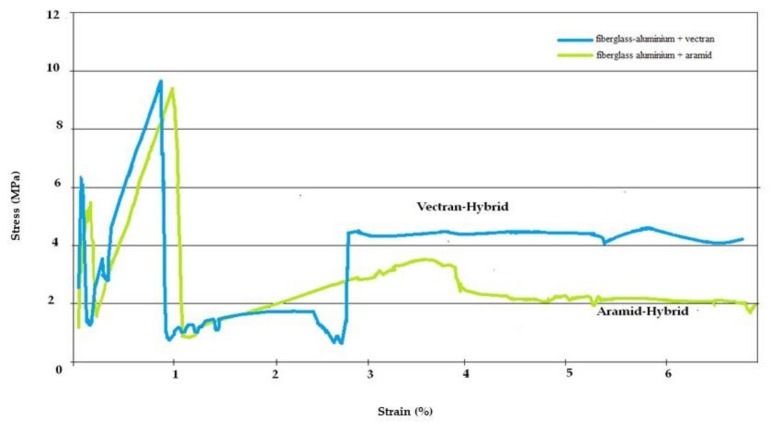
Stress–strain response of hybrid composite laminates during three-point bending testing.

The experimental results are shown in Figure 3, after the various composite samples were broken under three-point bending tests. These are plotted in terms of applied stress versus strain under the crosshead of the tester. All samples for the different composites had the same span length, allowing superimposition of the stress/strain plots for each group of samples.

As can be assessed from Figure 3, the behavior of composite laminate in this situation was bending stiffness, delamination and oscillations during the test until peak-load in the laminate (shown better in the initial bending stages). Little oscillations before peak-stress may be attributed either to vibrations of the supports, or to some defects in composite laminates, dents, and delamination in the top and bottom faces of laminate composite structures, as well as initiation of damage in the specimens.

The stress–strain curves can be divided into three regions: The first region, linear in appearance, refers to the elastic deformation of the composite laminate and reaches a local first maximum where first cracking appears. In the second region, after the stress reaches the maximum bending strength, a significant drop in peak-stress is observed in the composite laminate specimens, and this sudden drop can be attributed to the extensive lamina cracking and delamination. After the stress drop, the specimen continued to sustain the stress but never exceeded the previous peak-stress, since only the reinforcement carried the stress. This can be reflected in the third region of the curve, where a stress plateau is observed until reaching the final failure. The flexural strength of the material, defined as the maximum fiber stress at failure on the tension side, is higher for the fiberglass/alu– Vectran composite than in the fiberglass aramid one. Comparing the two hybrid composites used in this test, the fiberglass/alu–Vectran performed better, as seen in its high peak-load, small damage area, and absence of delamination. Residual fracture strength for Vectran hybrids is at 42% of laminate yield strength, which suggests a high damage tolerance.

The calculated mechanical properties from three-point bend testing are shown in Table 2. The properties shown corroborate the above stated analysis for the superior response of the Vectran-based hybrid composites. Notably, they also show superior damage tolerance by exhibiting a higher residual fracture stress after the main peak, as shown in Table 2.

### 3.2. Thermomechanical Testing

#### 3.2.1. Creep Time–Temperature Superposition (TTS) Testing

**Figure 4:**
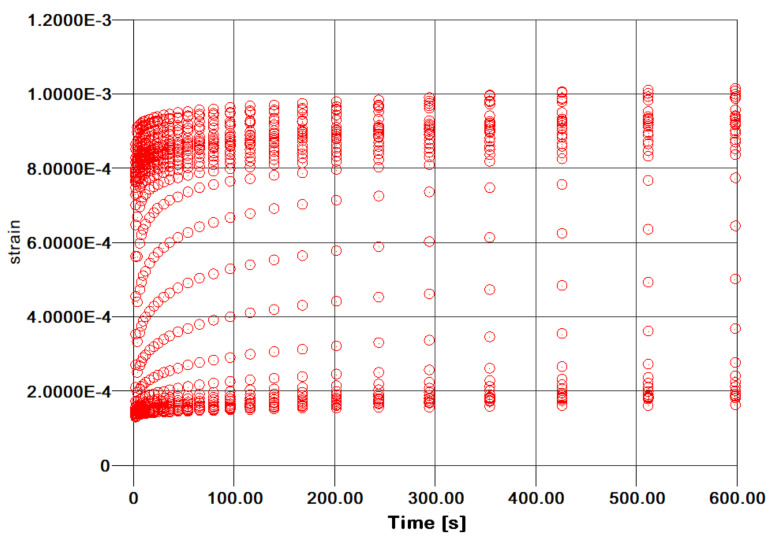
Kevlar/Aluminum–glass composite hybrid creep time–temperature superposition TTS curves.

Creep compliance testing was conducted at different stress levels so as to obtain a cloud of curves over time, as shown in Figure 4 for the Kevlar/aluminum–glass hybrid composite specimens. Evidently, the curves approximately show the self-similarity which is required to proceed to TTS modeling of the specimen’s creep response. The authors do not wish to refer analytically to TTS theory for polymers and composites, as it is given in the open literature in full detail. The TTS, or time–temperature superposition principle (TTSP), is widely used in the long-term characterization modeling for polymers [21,22,23] and composite materials [24,25,26], and also their stability response over aging effects [27,28].

**Figure 5:**
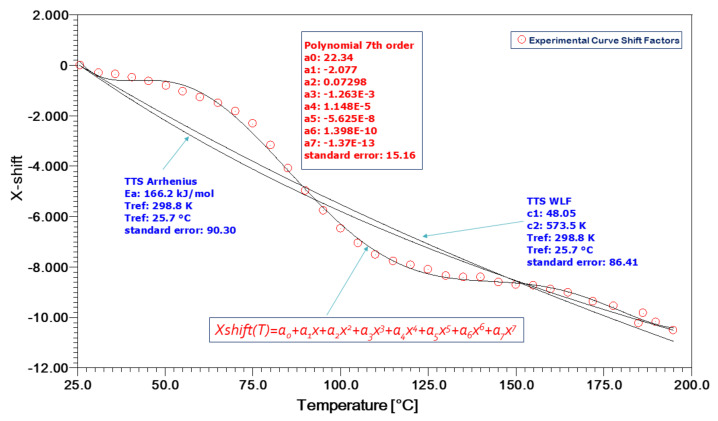
Kevlar/aluminum–glass composite hybrid creep TTS Shift factors a(T).

In Figure 5, the efforts to model the TTS experimental curve shift factors, as approached by the Arrhenius equation, as well as the William–Landel–Ferry (WLF) equations, are documented, as the arrows indicate. Evidently, this cannot be done for all types of shift factors as a function of temperature. Both the Arrhenius and WLF theories prescribe application above and below the glass transition temperature of the matrix polymer, respectively. Therefore, they are inapplicable in the regime near to the Tg (glass transition temperature) where, for example, the Kevlar/aluminum–glass hybrid composites exhibit a sigmoidal behavior of the shift factors with temperature. This behavior is impossible to model using the usual theories.

We have employed a 7th order polynomial equation with constant factors of the type:

(2) $$Xshift\left(T\right)=a_{o}+a_{1}x+a_{2}x^{2}+a_{3}x^{3}+a_{4}x^{4}+a_{5}x^{5}+a_{6}x^{6}+a_{7}x^{7}$$

This polynomial could very effectively model all types of X-shift curves and, moreover, it was actually observed that all curves were converging above the 3rd or 4th order very effectively.

**Figure 6:**
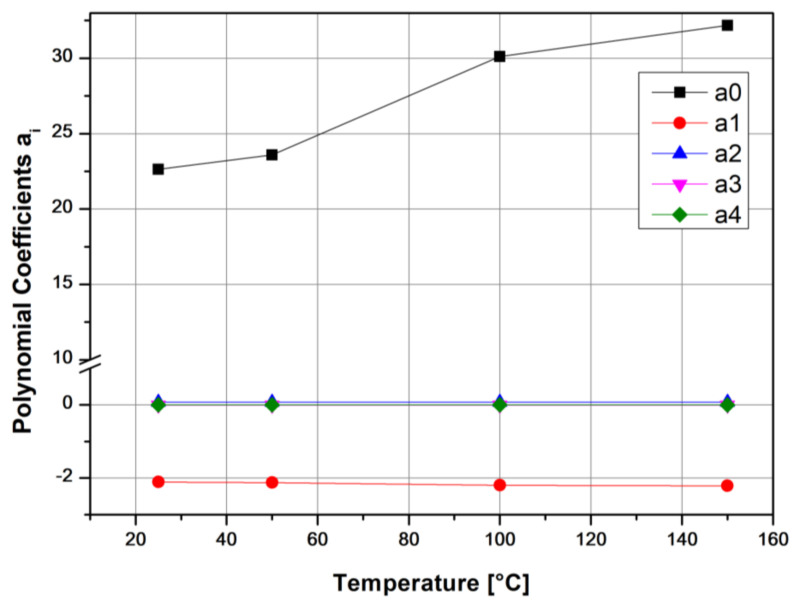
Kevlar/aluminum–glass composite hybrid creep, TTS, polynomial constants as a function of reference temperature Tref.

Thus, the model of Equation (2) was applied at four different reference temperatures; namely, 25, 50, 100 and 150°. Results obtained for all polynomial constants, as obtained by the approximation performed by the software Microcal ORIGIN 9.0, are given in Table 2. The first four constants of Equation (2) are plotted against reference temperature in Figure 6.

For this specific material, it can be seen that TTS response is largely dependent on the *α*_0_ polynomic factor and somewhat on the *α*_1_. This factor varies the most with temperature, which is much in contrast with the second-, third- and fourth-order factors.

**Figure 7:**
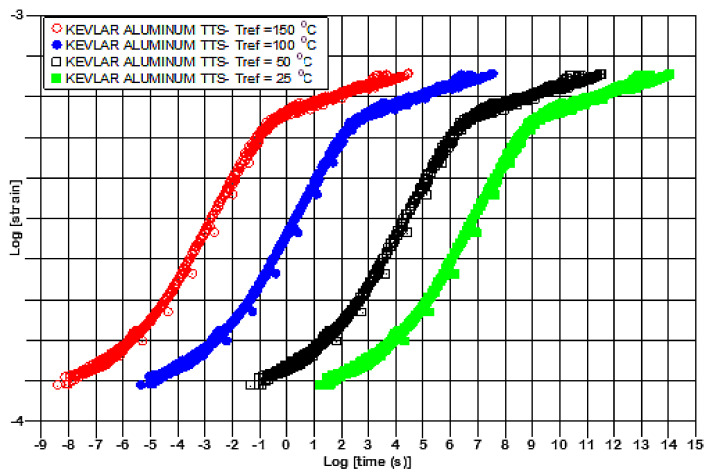
Kevlar/aluminum–glass composite hybrid, creep TTS master curves.

Finally, by using the nearly constant values of the second, third and fourth polynomic constants and the average value for *α*_0_ calculated from Table 3, namely *α*_0_ = 27,13, the creep-curves could be calculated, as shown in Figure 7 for three different reference temperatures. The curves are perfectly self-similar, as shown in Figure 7, and this opens new areas for novel modeling of the TTS response of polymers and composites.

**Figure 8:**
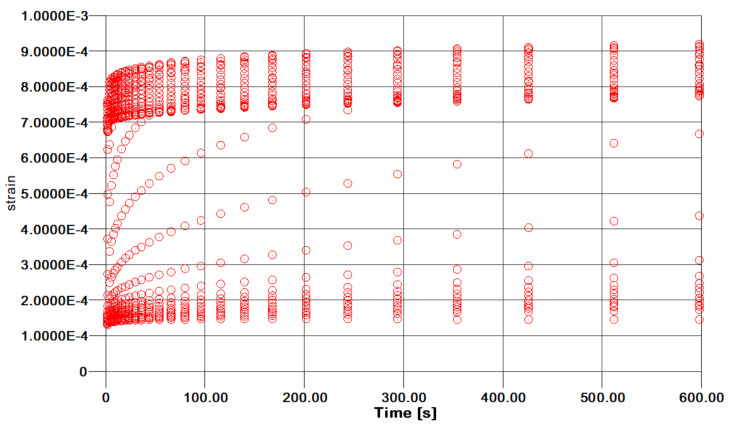
Vectran/aluminum–glass composite hybrid creep TTS curves.

**Figure 9:**
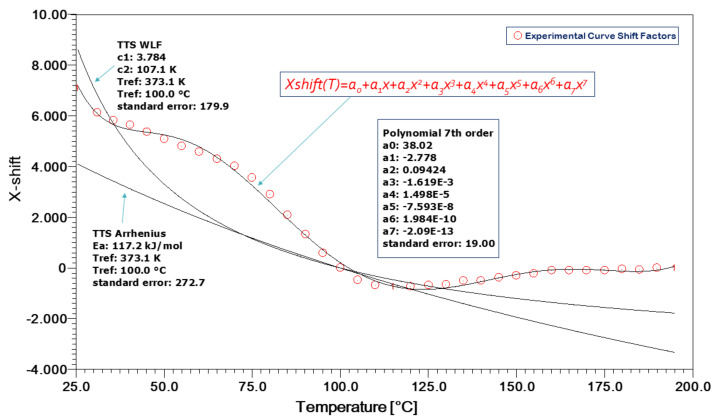
Vectran/aluminum–glass composite hybrid shift factors *α*(T).

The same procedure was repeated for the Vectran/Aluminum–glass composite hybrids. The specimen creep response was obtained for different stress levels, as shown in Figure 8. Afterwards, the shift factors of the experimentally obtained curves were plotted, as seen in Figure 9. The sigmoidal behavior of the experimental TTS data for the curve shift factors disallows the application of the usual Arrhenius and WLF models, respectively. Again, a 7th-order polynomial equation (as in Equation (1)) was employed as shown in Figure 9. This approach delivered proper results with respect to the X-shift factor modeling as a function of temperature. Practically, the 4th order was enough to approach the sigmoidal curve.

**Figure 10:**
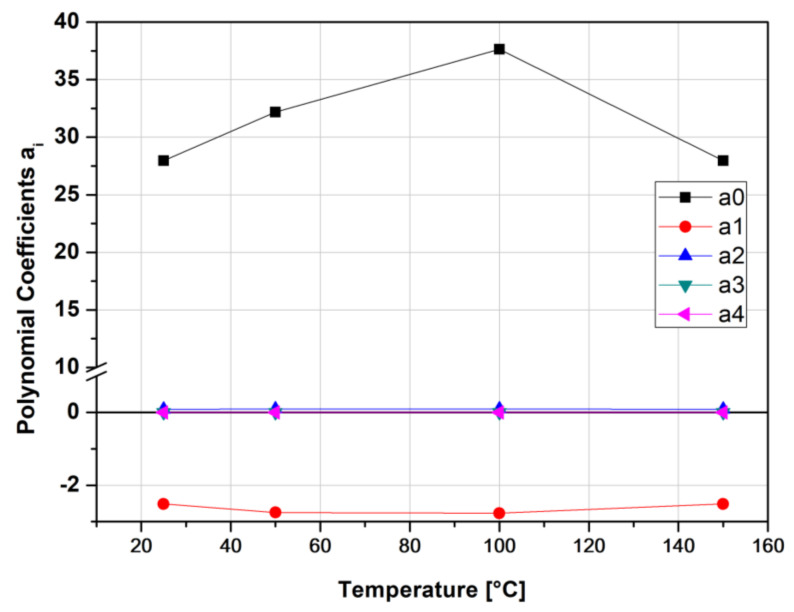
Vectran/aluminum–glass composite hybrid, polynomial constants as a function of reference temperature Tref.

**Figure 11:**
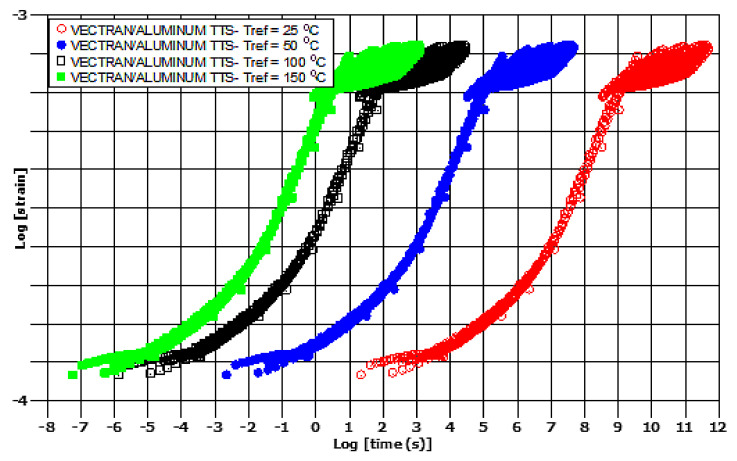
Vectran/aluminum–glass composite hybrid, creep TTS master curves.

The polynomial constants for the first four orders are shown in Figure 10, as a function of temperature, whereas in Table 3, these are presented up to the seventh order. Again, from the related Table 3, and Figure 10, the *α*_0_ polynomic factor, and to some extent the *α*_1,_ exhibit the largest variations in temperature. By using the results from Table 3, considering the almost constant values of the *α*_1_, *α*_2_, *α*_3_, polynomic factors and for the mean value of *α*_0_ = 31.43 the Master Curves for creep TTS could be constructed, as shown in Figure 11.

The curves are, again, almost perfectly self-similar, with the exception of a diffuse cloud of data, which all curves exhibit at the high-strain–high-time regime. From our own experience, these are associated with instabilities in obtaining the experimental data, not visible in the data collection phase.

Of course, the methodology presented here for obtaining valid TTS X-shift factors at almost all reference temperatures requires further investigation, and many more materials should be tested. The most interesting of these appears to be the further modeling of the viscoelastic physico-mathematical response of the polynomial constants *α_ι_*. As shown in Figure 6, Figure 7, Figure 8, Figure 9, Figure 10 and Table 3 and Table 4, respectively, they are not constants, but at least *α*_0_, appears to be a power function of temperature, whereas the *α*_1_ constant can be approached by a linear equation. More intense work and round-robin testing is required in order to accomplish this task, however.

#### 3.2.2. Dynamic Mechanical Analysis (DMA)

**Figure 12:**
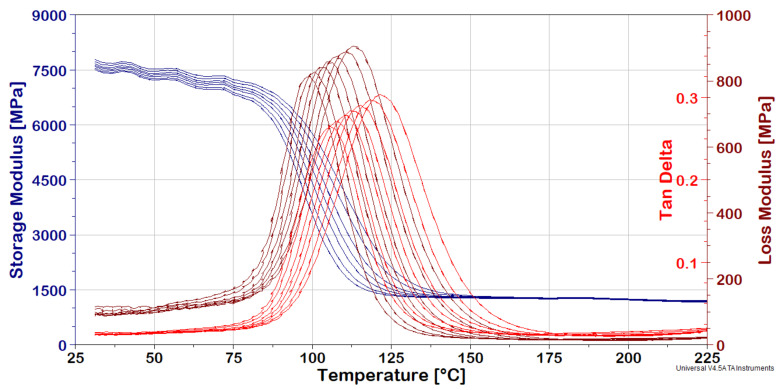
Kevlar/aluminum–glass composite Hybrid, DMA frequency-temperature scans.

**Figure 13:**
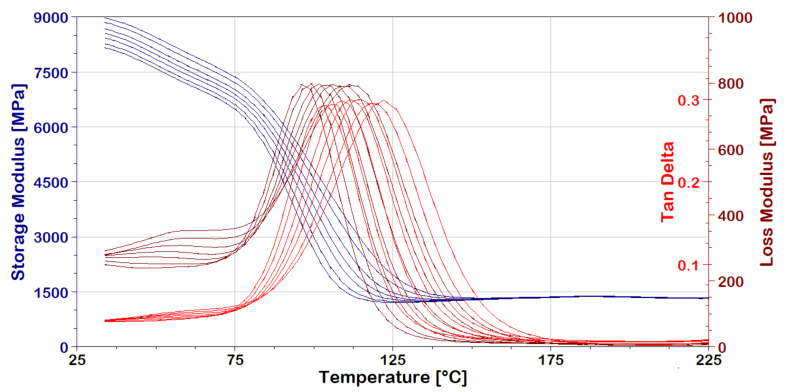
Vectran/aluminum–glass composite hybrid, DMA frequency-temperature Scans.

DMA spectra are shown in Figure 12 and Figure 13 for the Kevlar/aluminum and Vectran/aluminum–glass composite hybrid specimens, respectively. As already mentioned, they present the dynamic viscoelastic constants of the materials over a given range of temperatures and frequencies.

The effect of the Tg is very prominent on the abrupt fall in storage moduli (E’) at around 90–100 °C for both composites. This is a logical effect, since both are based on the same epoxy polymer matrix. Moreover, the peaks in loss moduli (E’’) and tan delta (tanδ) shift with frequency increases are traversing to the right, as expected. The effect is also observed for E’ plateaus but is less visible due to the absence of a related peak. Measurements cease to have meaning, as shown in Figure 12 and Figure 13, after 150–160 °C due to the extreme relaxation effect of the matrix resin.

### 3.3. Scanning Electron Microscopy Analysis

**Figure 14:**
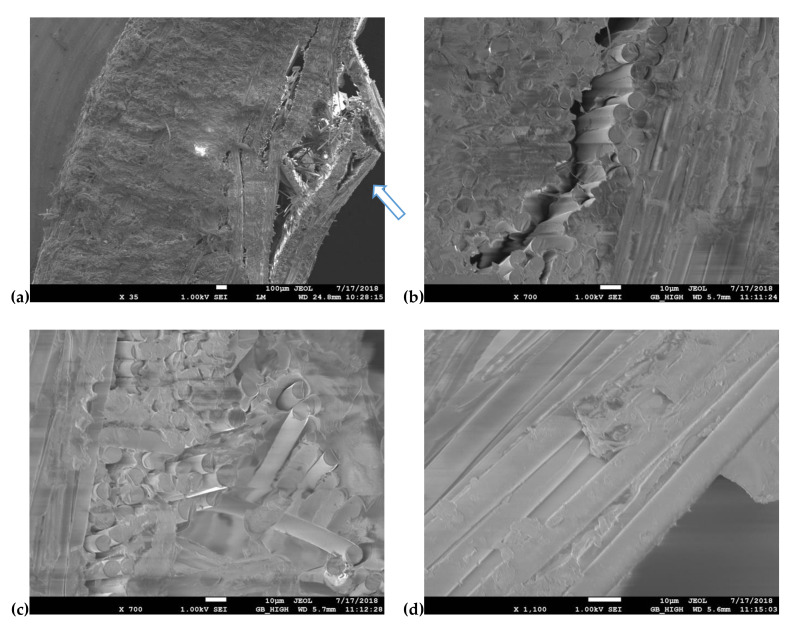
Scanning Electron microscopy from the fracture area of the Vectran/aluminum–glass composite hybrid three-point bending specimens (compressive side). (**a**) Compressive side, (**b**) delamination crack, (**c**) glass and aluminum fibers failure, (**d**) aluminum fibers exposed surfaces.

Fracture Surfaces (across the thickness direction) of Vectran/aluminum–glass composite hybrid three-point bending specimens were examined under the SEM. These specimens failed clearly in the compressive loading side of the composite. The compressive side was consistently the aluminum–glass composite layers side in bending tests (which arrows indicate). No failure was observed on the Vectran layers’ side (tensile side in bending). Multiple layer delamination and lamina fractures are seen very clearly in Figure 14a,b. On the other hand, aluminum fibers are well embedded in the polymer matrix, even though they are plastically deformed, as seen in Figure 14c, and especially good fiber wetting can be deduced from Figure 14d.

**Figure 15:**
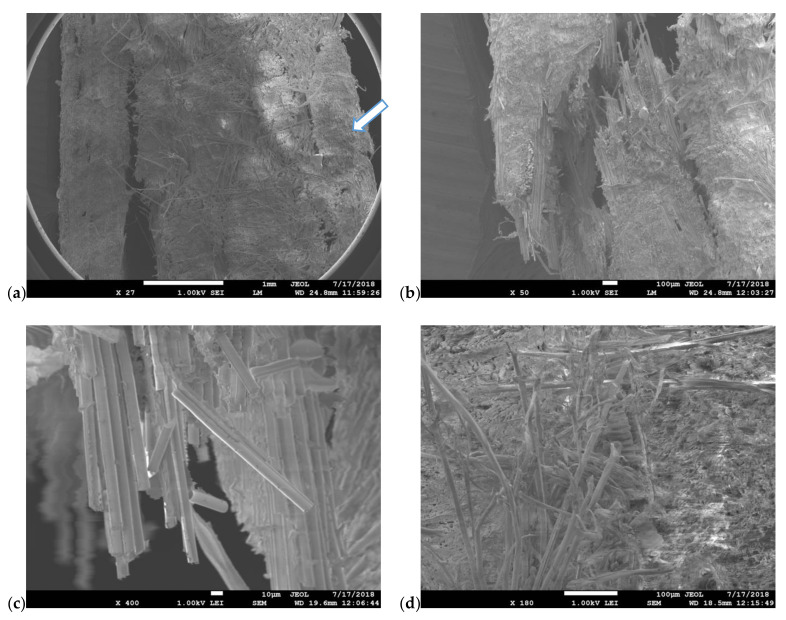
Scanning electron microscopy from the fracture area of the Kevlar/aluminum–glass composite hybrid three-point bending specimens (tensile side). (**a**) Tensile (left) and compressive side (arrow shows) delaminations, (**b**) delamination and ply cracking on the tensile side, (**c**) multiple glass fiber fragmentations, (**d**) brooming of Kevlar fibers.

SEM analysis has revealed the failure modes of the Kevlar/aluminum–glass composite hybrid three-point bending specimens. As we have seen, the tensile side in this case dominates the failure behavior for these composites. Delamination of the top layers is prominent (within Figure 15a,b this is easily deduced, where some delamination is also observed on the compressive side of the specimen, which the arrow indicates). Intra-ply failure for the glass-aluminum fabric layers is prominent in Figure 15b and multi-fractured glass fibers are seen in Figure 15c. Fiber brooming was also extensively observed on the tensile side for the Kevlar fibers, due to their inherent increased ductility in comparison to the glass fibers of the same hybrid fabric. The fracture behavior of these laminates can be explained by the fact that, as shown form bending tests, the Vectran plies allow for bending stresses to be retained at relatively higher levels after maximum bending strength has been reached. Therefore, larger bending strains can be further attained, which of course implies that the compressive side plies of aluminum–glass will be devastated due to exposure to higher bending strains than the Kevlar hybrids. This is demonstrated in Figure 14a.

## 4. Conclusions

Hybrid composite specimens were manufactured with Kevlar, aluminum/glass fibers and Vectran fiber plies.

The three-point-bending test behavior of the composite laminates was investigated. Three-point bending results showed that the hybrid composite laminates exhibited a progressive failure mode consisting of fiber failure, debonding (splitting), and delamination. The composite laminates exhibited a highly nonlinear stress–strain curve due to compressive yielding. High damage tolerance was observed for the Vectran hybrid specimens after the maximum bending strength was reached.

Fractography analysis performed by SEM showed that the Kevlar/aluminum–glass fiber composite hybrid specimens suffered from tensile failure under bending, accompanied by intra-ply failure. The Vectran textile reinforced hybrid specimens, on the other hand, exhibited compressive side failure, accompanied by multiple delamination and ply fractures. Vectran plies allowed for high bending tensile stresses (>40% of composite yield strength) to be retained at higher strains, which inflicted an impressive failure landscape on the composite’s compressive side.

Thermomechanical analysis, accompanied by TTS, showed that it was possible to model the non-linear viscoelastic behavior of the creep specimens near the glass transition temperature by a fourth-order equation for the shift factors. Further experimental results are required, however, to extensively validate this proposal, and to perform more intense modeling on the polynomial constants, which appear to be functions of reference temperature.

## Figures and Tables

**Table 1:** Mechanical-properties of composite laminae.

Property	Aramid Fabric	Vectran Fabric	Fiberglass/Aluminum Fabric
Tensile strength (MPa)	2863	2860–3206	2750
Elastic modulus (Gpa)	67	64–72	75
Fracture strain (%)	3.7	3.3–3.7	1.8–3.2
Mass per unit of area (gr/m^2^)	460	200	200
Weave	Plain	Twill 2 × 2	Twill 2 × 2
Warp (ends(threads)/cm)	6.7	6	16.8
Weft (ends(threads)/cm)	6.7	6	12

**Table 2:** Three-point bending mechanical properties of the hybrid specimens.

Property	Fiberglass/Aluminum-Vectran Lamina	Fiberglass/Aluminum-Aramid Lamina
Elastic modulus (GPa)	11.75	15.30
Elastic fracture strain (%)	0.89	0.99
Yield strength (Mpa) (@max stress)	9.78	9.58
Residual fracture strain (%)	6.85	6.97
Residual fracture strength (Mpa)	4.16	1.99

**Table 3:** Polynomial simulation factors for the shift curve a(T) at different reference temperatures for the Kevlar/Aluminum–glass composite hybrid creep TTS specimens.

	25 °C	50 °C	100 °C	150 °C
*α*_0_	22.64	23.6	30.12	32.17
*α*_1_	−2.107	−2.123	−2.197	−2.214
*α*_2_	0.07417	0.07461	0.07703	0.07797
*α*_3_	−1.29 × 10^−3^	−1.29 × 10^−3^	−1.33 × 10^−3^	−1.36 × 10^−3^
*α*_4_	1.18 × 10^−5^	1.17× 10^−5^	1.21 × 10^−5^	1.24 × 10^−5^
*α*_5_	−5.80 × 10^−8^	−5.76 × 10^−8^	−5.9 × 10^−8^	−6.18 × 10^−8^
*α*_6_	1.46 × 10^−10^	1.43 × 10^−10^	1.50 × 10^−10^	1.56 × 10^−10^
*α*_7_	−1.45 × 10^−13^	−1.41 × 10^−13^	−1.50 × 10^−13^	−1.57 × 10^−13^

**Table 4:** Polynomial simulation factors for the shift curve *α*(T) at different reference temperatures for the Vectran/aluminum–glass composite hybrid creep TTS specimens.

	25 °C	50 °C	100 °C	150 °C
*α*_0_	27.97	32.18	37.63	27.97
*α*_1_	−2.515	−2.749	−2.77	−2.515
*α*_2_	8.56 × 10^−2^	0.09439	0.09489	0.08559
*α*_3_	−1.47 × 10^−3^	−1.64 × 10^−3^	−1.65 × 10^−3^	−1.47 × 10^−3^
*α*_4_	1.35 × 10^−5^	1.53 × 10^−5^	1.54 × 10^−5^	1.35 × 10^−5^
*α*_5_	−6.78 × 10^−8^	−7.86 × 10^−8^	−7.94 × 10^−8^	−6.78 × 10^−8^
*α*_6_	1.74 × 10^−10^	2.08 × 10^−10^	2.12 × 10^−10^	1.74 × 10^−10^
*α*_7_	−1.78 × 10^−13^	−2.23 × 10^−13^	−2.28 × 10^−13^	−1.78 × 10^−13^

## Data Availability

Data available on request from corresponding author.

## References

[1] Shen Q., Omar M., Dongri S. (2012). Ultrasonic NDE Techniques for Impact Damage Inspection on CFRP Laminates. J. Mater. Sci. Res..

[2] Kiran R., Vasudevan A., Pugazhendhi L. (2021). A review on different hybrid composites for aircraft structures. Mater. Today Proc..

[3] Bin H., Boyao W., Zhanwen W., Shengli Q., Guofeng T., Dezhen W. (2020). Mechanical properties of hybrid composites reinforced by carbon fiber and high-strength and high-modulus polyimide fiber. Polymer.

[4] Don C. (2021). Flexural behaviour of carbon and glass reinforced hybrid composite pipes. Composites.

[5] Siva R., Kesavaram B., Martin J.J., Mathiselvan G., Bantha N., Sangeetha M. (2020). Mechanical behavior of sisal and banana fiber reinforced hybrid epoxy composites. Mater. Today Proc..

[6] Rana R.S., Buddi T., Purohit R. (2021). Effect of SiC reinforcement on the mechanical properties of Kevlar fiber based hybrid epoxy composites. Mater. Today Proc..

[7] Ramesh V., Anand P. (2020). Evaluation of mechanical properties on Kevlar/Basalt fiber reinforced hybrid composites. Mater. Today Proc..

[8] Shaari N., Wahab M.F.A., Shaari N.S., Jumahat A. (2020). Unhole and open hole tensile properties of hybrid Kevlar/glass fiber polymer composites with different stacking sequence. Mater. Today Proc..

[9] Zeno M., Mahisham I., Mahadi M.F., Mhod Amin A.N., Ahmad S.I., Mahmud J. (2020). Deformation and failure behavior of hybrid composite laminates made of Glass Epoxy and woven Kevlar Epoxy. Mater. Today Proc..

[10] Nunes J., Pouzada A., Bernardo C. (2002). The use of a three-point support flexural test to predict the stiffness of anisotropic composite plates in bending. Polym. Test..

[11] (2007). Standard Test Method for Flexural Properties of Polymer Matrix Composite Materials.

[12] Hallet S. (2000). Three-point beam impact tests on T300/914 carbon-fibre composites. Compos. Sci. Technol..

[13] Azzam A., Li W. (2014). An experimental investigation on the three-point bending behavior of composite laminate. IOP Conf. Ser. Mater. Sci. Eng..

[14] Vargas G., Mujika F. (2011). Determination of in-plane shear properties by three-point flexure test of ±45° anti-symmetric laminates. Polym. Test..

[15] Ary Subagia I.D.G., Kim Y., Tijing L.D., Kim C.S., Shon H.K. (2014). Effect of stacking sequence on the flexural properties of hybrid composites reinforced with carbon and basalt fibers. Compos. Part B Eng..

[16] Belingardi G., Cavatorta M.P. (2006). Bending fatigue stiffness and strength degradation in carbon–glass/epoxy hybrid laminates: Cross-ply vs. angle-ply specimens. Int. J. Fatigue.

[17] Carbajal N., Mujika F. (2009). An experimental investigation on the three-point bending behavior of composite laminate. Polym. Test..

[18] de Baere I., van Paepegem W., Degrieck J. (2009). Comparison of different setups for fatigue testing of thin composite laminates in bending. Int. J. Fatigue.

[19] Fujihara K., Huang Z.M., Ramakrishna S., Hamada H. (2004). Influence of processing conditions on bending property of continuous carbon fiber reinforced PEEK composites. Compos. Sci. Technol..

[20] Tomita Y., Morioka K., Iwasa M. (2001). Bending fatigue of long carbon fiber-reinforced epoxy composites. Mater. Sci. Eng. A.

[21] Naya S., Meneses A., Tarrío-Saavedra J., Artiaga R., López-Beceiro J., Gracia-Fernández C. (2013). New method for estimating shift factors in time–temperature superposition models. J. Therm. Anal. Calorim..

[22] Ionita D., Cristea M., Gaina C. (2020). Prediction of polyurethane behaviour via time-temperature superposition: Meanings and limitations. Polym. Test..

[23] Fesko D.G., Tschoegl Ν.W. (2007). Time temperature superposition in thermorheologically complex materials. J. Polym. Sci. Part C Polym. Symp..

[24] Ljubić D., Stamenovic M., Smithon C., Nujkic M., Medo B., Putic S. (2014). Time: Temperature superposition principle: Application of WLF equation in polymer analysis and composites. Zaštita Mater..

[25] Nosrati N., Ahad Z., Samaneh S. (2020). Long-term creep behaviour of E-glass/epoxy composite: Time-temperature superposition principle. Plast. Rubber Compos..

[26] Ornaghi H., Almeida J.M., Monticeli F., Neves R., Cioffi M.D. (2020). Time-temperature behavior of carbon/epoxy laminates under creep loading. Mech. Time-Depend. Mater..

[27] Guedes R.M., Alcides S., Hugo F. (2007). Influence of moisture absorption on creep of GRP composite pipes. Polym. Test..

[28] Guedes R.M., Rui M. (2011). A viscoelastic model for a biomedical ultra-high molecular weight polyethylene using the time–temperature superposition principle. Polym. Test..

